# Highly stable tandem solar cell monolithically integrating dye-sensitized and CIGS solar cells

**DOI:** 10.1038/srep30868

**Published:** 2016-08-04

**Authors:** Sang Youn Chae, Se Jin Park, Oh-Shim Joo, Yongseok Jun, Byoung Koun Min, Yun Jeong Hwang

**Affiliations:** 1 Clean Energy Research Center, Korea Institute of Science and Technology, Hwarang-ro 14-gil 5, Seongbuk-gu, Seoul, 02792, Republic of Korea; 2 Department of Chemistry, College of Science, Korea University, 145, Anam-ro, Seongbuk-gu, Seoul, 02841, Republic of Korea; 3 Department of Materials Science and Engineering, Korea University, 145, Anam-ro, Seongbuk-gu, Seoul, 02841, Republic of Korea; 4 Department of Materials Chemistry and Engineering, Konkuk University, 120 Neungdong-ro, Gwangjin-gu, Seoul, 143-701, Republic of Korea; 5 Green School, Korea University, 145, Anam-ro, Seongbuk-gu, Seoul, 02841, Republic of Korea

## Abstract

A highly stable monolithic tandem solar cell was developed by combining the heterogeneous photovoltaic technologies of dye-sensitized solar cell (DSSC) and solution-processed CuIn_x_Ga_1-x_Se_y_S_1-y_ (CIGS) thin film solar cells. The durability of the tandem cell was dramatically enhanced by replacing the redox couple from 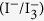 to [Co(bpy)_3_]^2+^ /[Co(bpy)_3_]^3+^), accompanied by a well-matched counter electrode (PEDOT:PSS) and sensitizer (Y123). A 1000 h durability test of the DSSC/CIGS tandem solar cell in ambient conditions resulted in only a 5% decrease in solar cell efficiency. Based on electrochemical impedance spectroscopy and photoelectrochemical cell measurement, the enhanced stability of the tandem cell is attributed to minimal corrosion by the cobalt-based polypyridine complex redox couple.

The development of solar cells with tandem architecture has attracted attention due to the possibility of overcoming the Shockley-Queisser limit of single junction devices^1^,^2^. The power conversion efficiency of tandem solar cells can be improved by mechanically stacking or monolithically integrating two or more sub-cells with complementary absorption characteristics^3^. Mechanically stacked architecture has the advantage of manufacturing simplicity, but it potentially suffers from optical loss due to the presence of superfluous substrate within the two sub-cells^4^. In this context, monolithically integrated tandem architecture is more suitable for the ultimate goal of a tandem device, which is to facilitate the efficient absorbance of a broader range of wavelengths. However, there are still many issues to be resolved before highly efficient monolithic tandem solar cells can be mass produced, such as lattice and bandgap matching, tunnel junction fabrication, and recombination layers^5^,^6^.

To date, various tandem structures have been suggested based on a combination of inorganic/inorganic, organic/organic, or inorganic/organic solar sub-cells. A world record efficiency of 37% has been achieved by triple-junction solar cells based on III–V compound semiconductor materials (the InGaP/GaAs/InGaAs tandem structure). In addition, amorphous and microcrystalline silicon (a-Si/μc-Si) based inorganic tandem cells have exhibited a solar cell efficiency of 13.6%^7^. Organic/organic triple-junction solar cells have also been successfully manufactured using different band-gap polymers, with a solar cell efficiency of 11%^8^. Various forms of inorganic/organic solar cells (known as hybrid tandem solar cells) have been studied, such as dye sensitized solar cells (DSSC)/Si, DSSC/GaAs, and perovskite/μc-Si^3^,^9^,^10^,^11^.

Models suggest that the optimal bandgap for tandem solar cells is 1.7 eV and 1.1 eV for the top and bottom cells, respectively. Copper chalcopyrite semiconductors – Cu(In, Ga)(S, Se)_2_ (CIGS) – are especially promising candidates for tandem cells because the band gap can be tuned from 1.0 to 2.4 eV in accordance with the composition ratios^12^. However, it is difficult to achieve high efficiency with monolithic CIGS/CIGS tandem cells due to damage to the sub-cell during construction of the top CIGS solar cell and the low efficiency of this sub-cell^13^.

In addition to tandem architecture involving similar classes of CIGS materials, substantially different types of single cells have also been combined with CIGS cells. Of these, a CIGS-based tandem solar cell constructed with a DSSC sub-cell is very promising because single junction sub-cells fabricated on individual glass substrates can be easily assembled by gluing. Liska *et al*. were the first to present a DSSC/CIGS tandem structure, demonstrating enhanced voltage and power conversion efficiency compared to single-junction solar cells^14^. However, the rapid corrosion of the p-n junction by the iodide electrolyte created a serious stability issue. To overcome this problem, a ZnO/TiO_2_ protection layer on the CIGS sub-cell was applied, but it did not sufficiently resolve the problem^15^. The soft deposition (e.g., arc-plasma deposition) of Pt on the CIGS sub-cell to minimize the damage of the pre-made films during the fabrication of Pt catalyst film was also attempted, but the instability problem remained^16^.

Based on the results of previous studies, the major underlying cause of DSSC/CIGS tandem cell instability is the corrosive iodide-based electrolyte^4^. As such, a cobalt complex based redox electrolyte would be a promising candidate as a substitute because it has been proven to be much less corrosive to the metallic conductors^17^ of DSSC single cells. In addition, the redox potential of Co^2+^/Co^3+^ is more negative than that of an iodide redox couple, leading to higher open circuit voltage (V_oc_)^18^,^19^. In this study, by introducing a [Co(bpy)_3_]^2+^/[Co(bpy)_3_]^3^ redox couple and Y123 organic dye as a sensitizer, we produce a highly stable DSSC/CIGS tandem solar cell. Furthermore, we apply PEDOT:PSS as the cathode material onto the Al-doped zinc oxide (AZO) window layer of the bottom CIGS cell instead of the Pt catalyst layer, which provides additional stability in the tandem cell. In addition, in order to realize low-cost and printable tandem solar cells, the CIGS sub-cell was fabricated using a solution-processed synthetic method. A 1000 h durability test of the proposed DSSC/CIGS tandem solar cell in ambient conditions produced a 5% decrease in solar cell efficiency, which is a significant improvement on iodide electrolyte based cells.

## Results and Discussions

**Figure 1:**
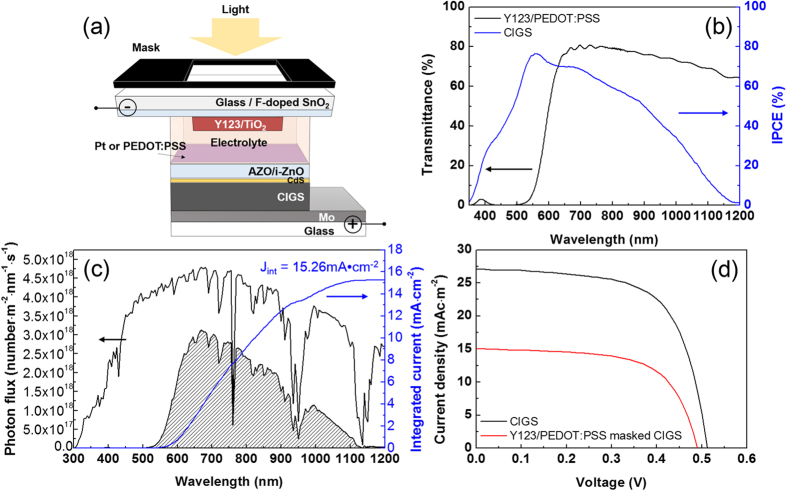
(**a**) Schematic diagram of a DSSC/CIGS tandem solar cell. (**b**) Transmittance of Y123-sensitized, TiO_2_/PEDOT:PSS-coated FTO glass and the IPCE value of a CIGS single junction solar cell. (**c**) Photon flux of one sun irradiation (black line), transmitted photon flux from Y123-sensitized, TiO_2_/PEDOT:PSS-coated FTO glass (filled black line), and intergraded photocurrent (blue line). (**d**) I-V curve of a single CIGS solar cell with and without a mask.

Considering the light absorption properties of each layer, we designed a tandem cell with a DSSC sub-cell on the top and a CIGS sub-cell on the bottom, as shown in Fig. 1a. For the top DSSC sub-cell, Y123 organic dye was used as a sensitizer. The HOMO-LUMO gap of Y123 is known to be 2.0 eV, which is ideal for the efficient absorption of shared light with the bottom cell in tandem architecture because the band-gap of the bottom CIGS absorber film is 1.1 eV. To gather more information regarding the efficiency of the tandem device design, the incident light distribution toward DSSC and CIGS sub-cells was investigated. Figure 1b shows the transmittance spectra of the top DSSC sub-cell with Y123 dye-sensitized TiO_2_ film and a PEDOT:PSS cathode, from which the actual incident light arriving at the bottom CIGS sub-cell can be estimated when the DSSC/CIGS tandem cell is used under a photon flux of 1 Sun irradiance (AM 1.5 filter, 100 mW∙cm^−2^ intensity; see the solid black line in Fig. 1c). The integrated short circuit current (J_int, bottom cell_, 15.26 mA∙cm^−2^) of the CIGS solar cell was calculated using equation (1)^20^, which combines the actual incident light and the incident photon to current conversion efficiency (IPCE) of the CIGS cell (Fig. 1b).


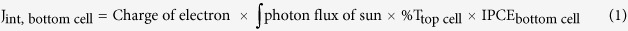


This matches well with the measured short circuit current (J_sc_, 15.1 mA∙cm^−2^) of the CIGS single cell with the dye-sensitized TiO_2_/PEDOT:PSS (Fig. 1d). In the DSSC/CIGS tandem cell configuration, the estimated J_sc_ values of the integrated bottom CIGS sub-cell are higher than the typical J_sc_ values of the top DSSC sub-cells, which are below 10 mA∙cm^−2^ (Table 1). This implies that the bottom CIGS sub-cell does not limit the photocurrent of the series-connected tandem cell.

**Figure 2:**
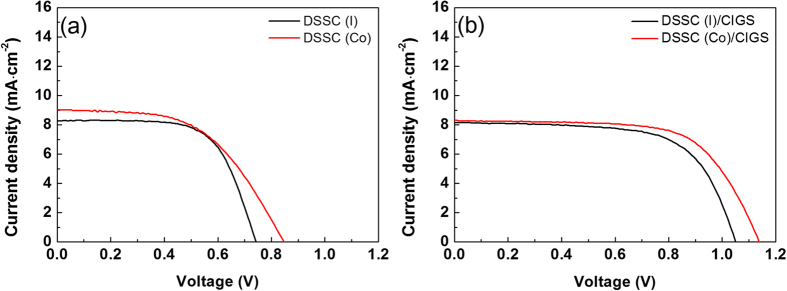
I–V graphs of single DSSC or DSSC/CIGS tandem solar cells. (**a**) DSSCs with different electrolytes and (**b**) DSSC/CIGS tandem cells with electrolytes containing iodine based (I) and cobalt based (Co) redox couples.

The current-voltage (I–V) characteristics of the single DSSC and CIGS solar cells and the tandem solar cell were measured (Figs 1d and 2); their performance is summarized in Table 1. In previous research, tandem DSSC/CIGS cells were constructed based on the commonly used redox couple electrolyte, 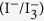 in acetonitrile. However, due to the corrosive nature of this electrolyte, the CIGS sub-cells were rapidly destroyed, resulting in the significant reduction of solar cell efficiency. In order to enhance the stability of this form of tandem cell, a cobalt complex was applied to the electrolyte in this study. This cobalt complex based electrolyte is known to be efficient when the PEDOT:PSS counter electrode was used instead of the traditional Pt film. (Hereafter, a DSSC with a cobalt complex electrolyte and a PEDOT:PSS counter electrode is referred to as DSSC(Co)). The DSSC(Co) exhibited a higher V_oc_ compared to the cell using an iodine electrolyte and Pt counter electrodes – hereafter called DSSC(I) – due to the more positive redox potential^19^ of [Co(bpy)_3_]^2+^/[Co(bpy)_3_]^3+^ than that of 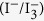 (Fig. 2a).

When an DSSC sub-cell was connected with an CIGS solar sub-cell in a series configuration, the V_oc_ of the tandem cell increased by ~300 mV from the V_oc_ of the DSSC single cell, while its J_sc_ was similar to that of the DSSC single cell (Fig. 2b). The DSSC(Co)/CIGS tandem cells demonstrated a ~100 mV larger V_oc_ than the DSSC(I)/CIGS tandem cells with similar J_sc_ values. The highest power conversion efficiency (6.11%) was obtained from DSSC(Co)/CIGS tandem cells. However, the V_oc_ of the tandem cells was 200 mV lower than the ideal voltage, which is the sum of each individual single cell. According to the J-V characteristics in Fig. 1d, the V_oc_ of masked CIGS solar sub-cells was found to decrease by only ~20 mV even though a large proportion of the incident light was intercepted by the top DSSC cells. Thus, the main loss is assumed to be caused by the series connection resistance of the tandem device.

**Figure 3:**
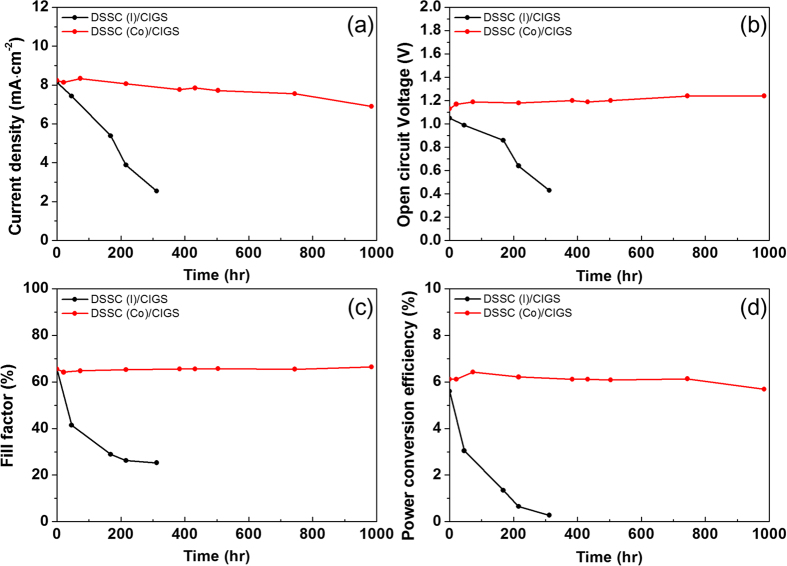
Solar cell parameters for DSSC/CIGS tandem cells during 1000 h testing: (**a**) current density, (**b**) open circuit voltage, (**c**) fill factor, and (**d**) power conversion efficiency.

To investigate the influence of the redox electrolyte on the durability of the DSSC/CIGS tandem cell, solar cell performance was monitored for 1000 h in an ambient environment (i.e., with no irradiation). Figure 3 shows the changes in the parameters of each device with aging time. After 50 h, both the fill factor and the power conversion efficiency of the DSSC(I)/CIGS tandem cell drastically decreased. Meanwhile, the DSSC(Co)/CIGS tandem cell had significantly enhanced stability over the course of the 1000 h test. In particular, the DSSC(Co)/CIGS cell demonstrated nearly identical to initial solar cell performance until 500 h. The 5% reduction in power conversion efficiency after 1000 h is an indicator of the role of the redox couple electrolyte in the stability of the DSSC/CIGS tandem cells.

**Figure 4:**
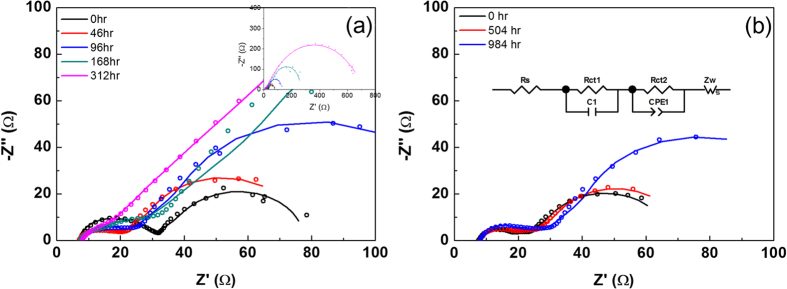
Nyquist plot of EIS spectra from DSSC/CIGS tandem cells by aging time. A DSSC/CIGS tandem cell with (**a**) an iodine-based electrolyte and (**b**) a cobalt-based electrolyte. Unfilled circles are measured values from the solar cell, and the solid lines were obtained from fitting data with equivalent circle, an inset of (**b**).

In order to further understand the degradation of the tandem cell, electrochemical impedance spectroscopy (EIS) was measured for each tandem cell type in relation to aging time (Fig. 4 and Table 2). An equivalent circuit^21^ consists of the series resistance of the solar cell (R_s_), charge transfer resistance, and the constant phase element between the redox couple catalyst-CIGS composite and the electrolyte (R_ct1_, CPE_1_), the charge transfer resistance and double layer capacitance between the dye-sensitized TiO_2_ and the electrolyte (R_ct2_, CPE_2_), and the Warburg diffusion element (Z_w_) related to the diffusion of the electrolyte (see inset of Fig. 4b). Differences in EIS parameter changes were observed for the redox couples 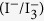 and [Co(bpy)_3_]^2+^/[Co(bpy)_3_]^3+^. R_s_ and R_ct2_ did not exhibit much difference during the running time, but R_ct1_ and the Warburg coefficient (R_w_) gradually increased, especially in the solar cells with the iodine-based redox couple. For instance, R_ct1_ increased from 31.53 to 555.6 Ω after 312 h. It is thought that the corrosive iodine species may lead to the deterioration of the Mo/CIGS/CdS/i-ZnO/AZO structure (the p-n junction) or the Pt catalysts on AZO^14^,^15^. Since the R_ct1_ values did not change before 312 h, we believe that the Pt catalysts on AZO were not severely damaged until this time. However, R_w_ and shunt resistances constantly decreased from the beginning of solar cell operation (Fig. 3c). Therefore, the main reason for the drop in cell performance could be p-n junction deterioration. Note that the DSSC single cell which used an iodine-based electrolyte was very stable until 500 h (Figure 1S). When this iodine redox couple is replaced with a Co-based electrolyte, the DSSC(Co)/CIGS tandem cell showed no noticeable change in R_ct1_ before 1000 h. Therefore, the chemical species of the redox couple is a key factor in the manufacture of highly stable DSSC/CIGS tandem cells.

The decrease in the power conversion efficiency of the DSSC(Co)/CIGS cell may also be related to the Warburg diffusion element. Therefore, we focused on the change of the Warburg diffusion element (Z_w_), which is expressed in equation (2)^22^.


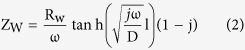


where ω, j, D, 1 and R_w_ are the angular frequency, the imaginary part of the complex, the diffusion coefficient of the redox species, the Nernst diffusion layer thickness, and the Warburg coefficient, respectively. R_w_ is expressed in equation (3) as follows:


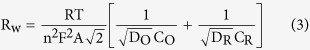


where R, T, n, F and A denote the ideal gas constant, the temperature, the number of electrons, the Faraday constant, and the surface area of the electrode, respectively. D_O_ and D_R_ is the diffusion coefficient of the oxidizing species and reducing species, respectively, and C_O_ and C_R_ is the concentration of the redox species on each surface. According to equation (3), an increase in R_w_ indicates a mass transport problem for the redox couple in an electrolyte. The concentrations of redox species affect changes in R_w_ over the time of operation because most of the components in R_w_ are constants. For cobalt-based redox couples, the bulk concentrations of [Co(bpy)_3_]^2+^ and [Co(bpy)_3_]^3+^ were 0.22 and 0.033 M, respectively. Since bulk concentrations did not change over the running time, a decrease in the surface concentration of [Co(bpy)_3_]^3+^ lead to an increase in R_w_. [Co(bpy)_3_]^3+^ can diffuse into ZnO or CIGS crystals in the sub-cell through grain boundaries which do not have catalytic active sites; therefore, the reduction rate of [Co(bpy)_3_]^3+^ will be slower compared to that on the PEDOT:PSS counter electrode. In other words, because [Co(bpy)_3_]^3+^ diffused into grain boundaries cannot contribute to the redox shuttle reaction, the effective surface concentration of [Co(bpy)_3_]^3+^ on the counter electrode will be decrease and consequentially increase in R_w_.

The R_ct1_ (the charge transfer resistance between the electrolyte and counter electrode) and the R_w_ of DSSC single cells which used an iodine-based electrolyte and a Pt catalyst on an FTO substrate were also investigated with 500 h of testing (Figure 2S). There was no corrosion of the platinized FTO counter electrode due to the iodine-based electrolyte, hence R_ct1_ did not increase. There was also no noticeable cracks or chinks in the platinized FTO surface and R_w_ did not increase over the course of the 500 h. This result supports our conjecture that the change in the surface concentration of the redox couple leads to a mass transport problem in electrolyte.

The reduction of power conversion efficiency due to the mass transport problem arising from decreasing surface concentration of [Co(bpy)_3_]^3+^ has also been suggested by Jiajia Gao *et al*.^23^. They investigated the stability of DSSCs with Y123 dye and cobalt-based redox couples and also observed an increase in the Warburg coefficient during 1000 h stability testing. We also speculate that the decrease in the surface concentration of iodide ions is due to the penetration of the iodide ions into the crystal boundaries of ZnO or CdS/CIGS, but this phenomenon is difficult to distinguish from the effect of corrosion by iodine-based electrolytes.

**Figure 5:**
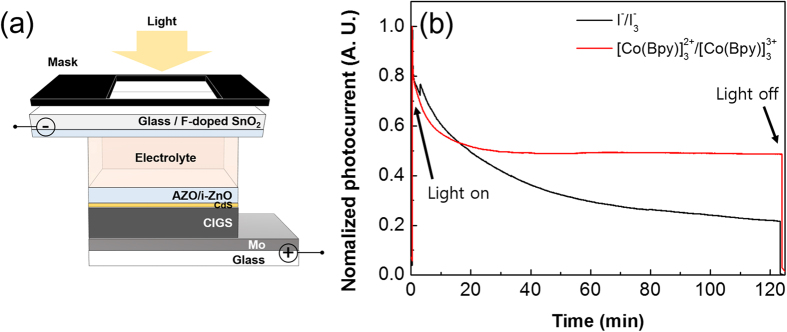
(**a**) Schematic diagram of a photoelectrochemical cell. (**b**) Current – Time (I-t) graph for a photoelectrochemical cell with different redox couple under light illumination.

To clarify the effect of corrosion by the redox couple on the CIGS p-n junction, we prepared photoelectrochemical (PEC) cells with iodine or cobalt complex based electrolytes with a CIGS p-n junction (Fig. 5a). Similar to the tandem cell structure, a PEC cell consisted of an AZO/i-ZnO/CdS/CIGS/Mo bottom cell, but the top cell had a bare FTO substrate, not a dye-sensitized TiO_2_ photoanode. In addition, a bare CIGS bottom cell without a catalyst film such as Pt or PEDOT:PSS was used in the PEC cell to examine whether the chemical species of the redox electrolyte influenced the stability of the CIGS bottom cell. A spike in the photocurrent-elapsed time curve may be observed because of the electron-hole pair recombination due to slow reduction/oxidation rate of redox couples without photoanodes or catalyst films^19^,^24^. In contrast to PEC cells containing an iodine-based redox couple, whose photocurrent continuously decreased, the photocurrent plateaued when cobalt complex based redox couples were used. This indicates that the reduced performance of the tandem device may be the result of the degradation of the CIGS solar cell due to corrosion by the redox couple. This view is supported by the increasing R_ct1_ values when the iodine-based redox couple was used in the tandem device.

## Conclusions

We successfully fabricated a monolithic tandem cell with a top Y123 dye-sensitized solar sub-cell and a bottom CIGS thin film solar sub-cell. To enable the production of low-cost, printable tandem solar cells, the CIGS sub-cell was fabricated by the solution-processed synthetic method. The tandem device demonstrated a high V_oc_ of up to ~1.1 V, with a J_sc_ that was limited by the photocurrent of the DSSC sub-cell in the series tandem configuration. The efficiency decreased by only 5% after 1000 h testing in ambient conditions when using the cobalt complex based electrolyte because of its low corrosiveness.

## Method

### Dye sensitized TiO_2_ thin film

Commercial TiO_2_ paste (D18-NT, Dyesol) was diluted by 18 wt% terpineol and 2 wt% ethylcelluose to control the pore size of the deposited film. A mesoporous TiO_2_ layer (4 μm) was prepared on a fluorine-doped tin oxide (FTO) glass (TEC8, Pilkington) using the screen printing method and was annealed at 500 °C. After annealing process, TiO_2_ film was kept in 0.04 M TiCl_4_ aqueous solution at 70 °C for 30 min followed by annealing at 500 °C in air. To sensitize the dye molecules, the mesoporous TiO_2_ film was immersed in 0.1 mM 3-{6-{4-[bis(2′,4′-dibutyloxybiphenyl-4-yl)amino-]phenyl}-4,4-dihexyl-cyclopenta-[2,1-b:3,4-b’]dithiophene-2-yl}-2-cyanoacrylic acid (Y123) in a 1:1 solution of acetonitrile (Sigma-Aldrich, 99.9%) and *tert*-butanol (Sigma-Aldrich, 99%) for 18 h.

### Preparation of redox couple electrolytes

Two types of redox couple electrolytes – an iodine based 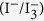 and a cobalt complex based electrolyte ([Co(bpy)_3_]^2+^/[Co(bpy)_3_]^3+^)– were tested. The commercial iodine-based electrolyte AN-50 (Solaronix) was used as the triiodide/iodide redox electrolyte. To prepare the cobalt complex based electrolyte, [Co(bpy)_3_](PF_6_)_2_ and [Co(bpy)_3_](PF_6_)_3_ were synthesized according to a previously reported method^25^, and then 0.22 M [Co(bpy)_3_](PF_6_)_2_, 0.033 M [Co(bpy)_3_](PF_6_)_3_, 0.4 M 4-tert-butylpyridine (Sigma-Aldrich, 96%), and 0.2 M LiClO_4_ (Sigma-Aldrich, 99.99%) were dissolved in acetonitrile (Sigma-Aldrich, 99.9%).

### Preparation of the CIGS film and solar cell fabrication

A CIGS solar cell was fabricated with a conventional Mo/CIGS/CdS/i-ZnO/AZO/Ni/Al structure. First, the Mo layer (~500 nm) was sputtered onto soda-lime glass using DC sputtering, and a solution-processed CIGS film was prepared on the Mo layer in accordance with previous research. A metal precursor mixture solution was prepared by dissolving an appropriate amount of Cu(NO_3_)_2_·xH_2_O (Sigma-Aldrich, 99.999%, 0.82 g), In(NO_3_)_3_∙xH_2_O (Sigma-Aldrich, 99.99%, 1.12 g), and Ga(NO_3_)_3_∙xH_2_O (Alfa Aesar, 99.999%, 0.41 g) in methanol (7.0 ml). After stirring for 30 min, polyvinyl acetate (PVA, Sigma-Aldrich, 1.0 g) in 10 ml methanol was added, and the mixture was stirred for another 30 min. The precursor mixture solution was spin-coated on the Mo-coated soda-lime glass substrate followed by annealing at 300 °C for 30 min; the coating and annealing steps were repeated six times to obtain the desired film thickness (~1.2 μm). The film was then selenized with elemental Se under H_2_S (1%)/Ar at 470 °C for 10 min in a quartz tube. Following this, a 60-nm-thick CdS buffer layer was deposited on 0.5 M KCN solution-treated CIGS film using chemical bath deposition (CBD), and i-ZnO (50 nm)/Al-doped ZnO (AZO; 500 nm) was deposited using the radio-frequency magnetron-sputtering method. A Ni (50 nm) and Al (500 nm) grid was deposited using electron beam evaporation as a current collector. The active area of the completed CIGS single cell was 0.245 cm^2^. In the DSSC/CIGS tandem solar cell, the current collector (Ni and Al grid) was not deposited.

### DSSC, DSSC/CIGS tandem cell, and photoelectrochemical cell fabrication

A sandwich-type DSSC was assembled with a Y123 dye-sensitized photoanode and a counter electrode using hot pressing with a thermoplastic spacer (Solaronix, Meltonix 1170-60). For the fabrication of a DSSC/CIGS tandem cell, an Mo/CIGS/CdS/i-ZnO/AZO structure was used instead of the counter electrode, as shown in Fig. 1a. Two different films (Pt and PEDOT:PSS) were compared for use as catalysts for redox couples on the counter electrode or the Mo/CIGS/CdS/i-ZnO/AZO structure. Pt (3 nm) was deposited using RF sputtering and a PEDOT:PSS solution was spin-coated and dried at 120 °C for 10 min. Finally, the iodine based or the cobalt complex based electrolyte was injected through a pre-drilled hole and sealed with a thermoplastic spacer. The active area of both the DSSC single cell and the DSSC/CIGS tandem cell was 0.25 cm^2^.

A photoelectrochemical (PEC) cell was fabricated following the same procedure as the DSSC/CIGS tandem solar cell, but a bare FTO substrate was used instead of a dye-sensitized photoanode.

### Characterization

Photovoltaic measurement of the single DSSC and CIGS solar cells, the tandem cell, and the photoelectrochemical cell was carried out with a potentiostat (Iviumstatpotentiostat, Ivium) under an AM 1.5 solar simulator which was equipped with a 300 W xenon lamp (ABET, Sun 2000) and an incident photon-to-current conversion efficiency (IPCE) measurement unit (PV measurement Inc.). The average solar cell performances were characterized with three samples for each type of solar cells. Stability tests were conducted under the same conditions as the photovoltaic measurement but the solar cells were kept in a dark environment while aging the cell. The transmittance of Y123 sensitized photoanodes was measured with a UV-Vis spectrometer (Varian, Cary 5000). Electrochemical impedance spectroscopy (EIS) was performed using open circuit potential and 1 sun simulated light illumination, with a frequency of 100 kHz to 0.1 Hz applied via a potentiostat (Iviumstatpotentiostat, Ivium). The obtained EIS spectra were fitted using Z-View software (ver. 2.8d).

## Additional Information

**How to cite this article**: Chae, S. Y. *et al*. Highly stable tandem solar cell monolithically integrating dye-sensitized and CIGS solar cells. *Sci. Rep.* **6**, 30868; doi: 10.1038/srep30868 (2016).

## Supplementary Material

Supplementary Information

## Figures and Tables

**Table 1:** Solar cell parameters.

Solar cell	J_sc_ (mA∙cm^−2^)	V_oc_ (V)	FF (%)	η^a^ (%)	η^b^ (%)
DSSC single cell (I)	8.28	0.74	66.1	4.05	4.02±0.47
DSSC single cell (Co)	9.04	0.85	53.2	4.09	4.34±0.31
DSSC/CIGS Tandem cell (I)	8.14	1.05	65.4	5.56	5.38±0.18
DSSC/CIGS Tandem cell (Co)	8.24	1.13	65.6	6.11	6.87±0.62
CIGS single cell	27.1	0.51	65.4	9.03	9.19±0.27
Masked CIGS	15.1	0.49	63.9	4.72	—

η^a^ is the median value of the power conversion efficiency, and η^b^ is a mean average value of the power conversion efficiency with a standard deviation (n = 3).

**Table 2:** Fitted values from the impedance spectra by aging time.

Solar cell (hr)	Rs (Ω cm^−2^)	R_ct1_ (Ω cm^−2^)	R_ct2_ (Ω cm^−2^)	R_w_ (Ω S^−1/2^∙cm^−2^)
DSSC (I)/CIGS (0)	31.53	35.18	46.96	196.4
DSSC (I)/CIGS (46)	29.97	22.04	20.97	230.7
DSSC (I)/CIGS (96)	31.48	29.59	23.50	407.6
DSSC (I)/CIGS (168)	30.86	24.88	31.37	953.6
DSSC (I)/CIGS (312)	32.98	555.6	21.25	2229.2
DSSC (Co)/CIGS (0)	32.78	18.18	33.98	180.1
DSSC (Co)/CIGS (504)	33.78	23.54	36.20	188.6
DSSC (Co)/CIGS (984)	34.56	29.96	42.88	344.6
