# Supplementary material for: Highly stable tandem solar cell monolithically integrating dye-sensitized and CIGS solar cells
Source: Sci Rep. 2016 Aug 4;6:30868. doi: 10.1038/srep30868 (PMC4973243; doi:10.1038/srep30868)
Supplement: Supplementary Information [file srep30868-s1.doc]

**Supplementary**

**Highly stable tandem solar cell monolithically integrating dye-sensitized and CIGS solar cells**

Sang Youn Chae^1,2,†^, Se Jin Park^1,3,†^, Oh-Shim Joo^1^, Yongseok Jun^4^, Byoung Koun Min^*,1,5^, and Yun Jeong Hwang^*^,**^1^**

^1^ Clean Energy Research Center, Korea Institute of Science and Technology, Hwarang-ro 14-gil 5, Seongbuk-gu, Seoul, 02792, Republic of Korea

^2^ Department of Chemistry, College of Science, Korea University, 145, Anam-ro, Seongbuk-gu, Seoul, 02841, Republic of Korea

^3^ Department of Chemical and Biological Engineering, Korea University, 145, Anam-ro, Seongbuk-gu, Seoul, 02841, Republic of Korea

^4^ Department of Materials Chemistry and Engineering, Konkuk University, 120 Neungdong-ro, Gwangjin-gu, Seoul, 143-701, Republic of Korea

^5^ Green School, Korea University, 145, Anam-ro, Seongbuk-gu, Seoul, 02841, Republic of Korea

**†**These authors contributed equally.

* Corresponding author : Yun Jeong Hwang and Byoung Koun Min, E-mail: [yjhwang@kist.re.kr](mailto:yjhwang@kist.re.kr), [bkmin@kist.re.kr](mailto:bkmin@kist.re.kr)


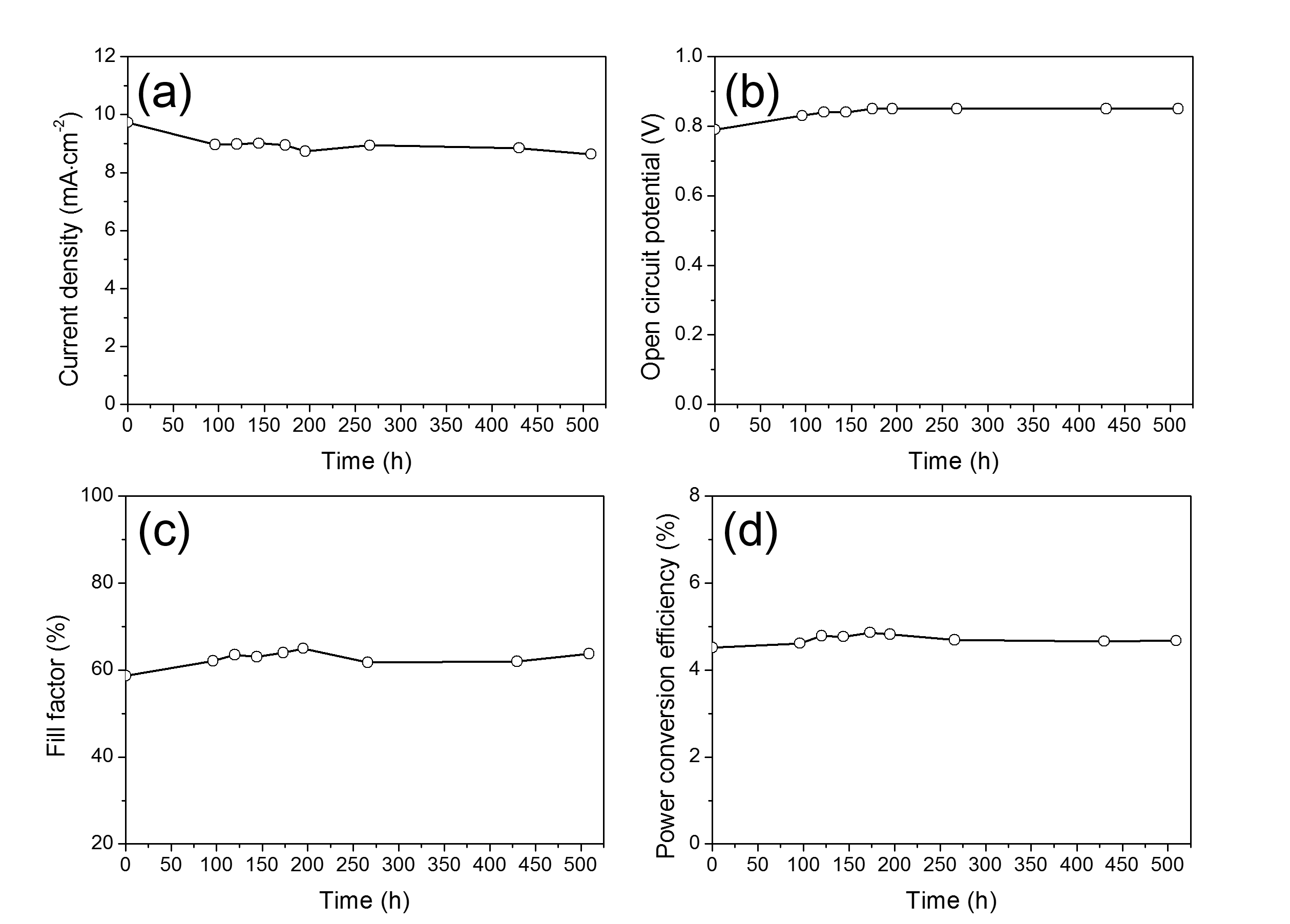


Figure 1S. Solar cell parameters of the
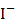
/
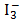
redox couple used DSSC single cells during 1000 h. Current density (a), open circuit potential (b), fill factor (c), and power conversion efficiency (d). Platinum was used for reduction catalyst for I_3_^-^.


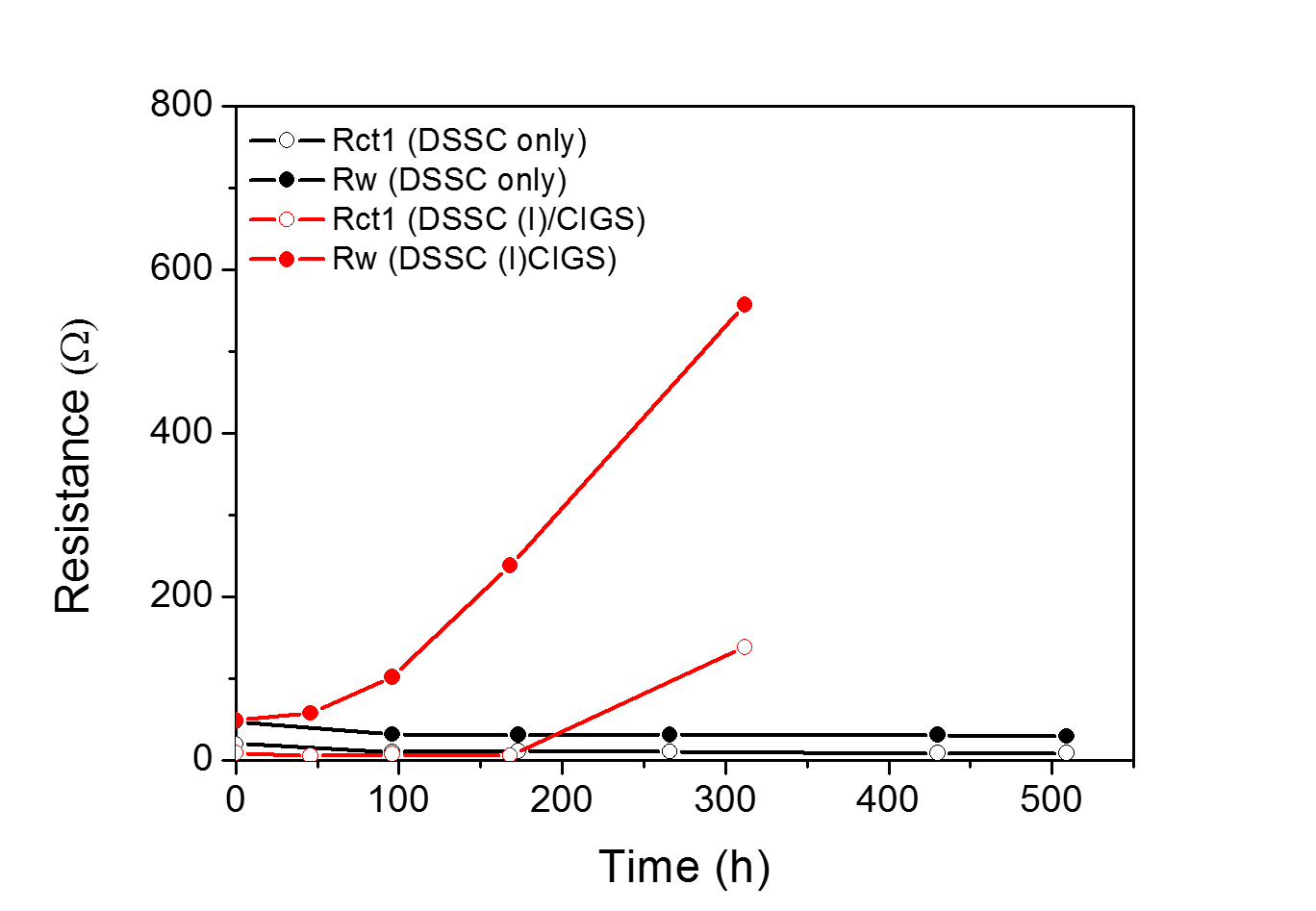


Figure 2S. Electrolyte/counter electrode-charge transfer resistance (unfilled circle) and Warburg coefficient (filled circle) of counter electrode in DSSC single cell or bottom cell surface in DSSC (I)/CIGS tandem cell. The
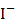
/
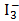
 redox couple, and Pt were used as the electrolyte and the counter electrode, respectively.
